# Detection of progression of asymptomatic severe aortic stenosis using Apple Watch–estimated maximal oxygen consumption (VO_2_ max): a case report

**DOI:** 10.1093/ehjcr/ytag246

**Published:** 2026-04-07

**Authors:** Mukhtar Al-Saadi, Aswin Srinivasan, Elizabeth Langlois, Pranav Loyalka

**Affiliations:** Department of Cardiology, HCA Houston/University of Houston College of Medicine, 1200 Binz St., Houston, TX 77004, USA; Department of Cardiology, HCA Houston/University of Houston College of Medicine, 1200 Binz St., Houston, TX 77004, USA; Structural Heart & Valve Center, Houston Heart, HCA Houston Healthcare Medical Center, The University of Houston Medical School, 1200 Binz St., Suite 900, Houston, TX 77004, USA; Structural Heart & Valve Center, Houston Heart, HCA Houston Healthcare Medical Center, The University of Houston Medical School, 1200 Binz St., Suite 900, Houston, TX 77004, USA

**Keywords:** Aortic stenosis, VO_2_ max, Apple Watch, Wearable device, Transcatheter aortic valve replacement (TAVR), Case report

## Abstract

**Background:**

Symptomatic aortic stenosis (AS) constitutes a class I indication for aortic valve intervention, either by surgical or transcatheter aortic valve replacement (TAVR). Progressive AS can reduce maximal oxygen consumption (MVO_2_), yet this parameter is not routinely evaluated in clinical practice.

**Case summary:**

We present a case of a 68-year-old with hypertension and hyperlipidaemia, initially diagnosed with moderate AS [aortic valve area (AVA) 1.3 cm^2^, mean gradient 23 mmHg, peak velocity 3.2 m/s]. He was asymptomatic and maintained a high level of physical activity, including treadmill running. Over the course of 3 years, the patient used an Apple Watch® to monitor estimated VO_2_ max. Despite stable exercise tolerance, his recorded VO_2_ max decreased progressively from 36 to 26 mL/kg/min. Concerned by the trend, he sought our evaluation. Repeat echocardiography showed severe AS (AVA 0.6 cm2, mean gradient 43 mmHg, peak velocity 4.29 m/s) with preserved left ventricular ejection fraction (LVEF 60%–65%). The patient subsequently underwent TAVR with a 26 mm Edwards Lifesciences Sapien 3 Ultra Valve®. At 1-year follow-up, his Apple Watch–derived VO_2_ max improved to 41 mL/kg/min. Echocardiography confirmed a well-seated prosthesis with a mean gradient of 9 mmHg.

**Discussion:**

This case illustrates the potential utility of consumer wearable technology (Apple Watch) for detecting progressive asymptomatic AS using estimated VO_2_ max feature. Continuous tracking of estimated VO_2_ max may serve as an adjunct parameter to guide timing of intervention in selected patients.

Learning points

- Progressive decline in wearable-estimated VO_2_ max may indicate progression of aortic stenosis even before patient becomes symptomatic.
- Consumer wearable technology can provide continuous physiologic data complementing standard clinical and echocardiographic follow-up.
- Interpretation of data should consider device limitations, physiologic variability, and lack of formal validation.

## Introduction

Aortic stenosis (AS) is the most common valvular heart disease. Severity and symptoms onset mark a critical transition point requiring valve replacement. However, many patients remain asymptomatic despite haemodynamically severe disease. Objective assessment of functional capacity such as maximal oxygen consumption (MVO_2_) can provide valuable information regarding disease severity and prognosis. Cardiopulmonary exercise testing is the gold standard to evaluate MVO_2_, but is not routinely performed in this population.

Apple Watch offers continuous, non-invasive monitoring of estimated VO_2_ max based on heart rate and motion sensors during physical activities. These data provide insights into the physiologic performance outside clinical settings. We present a case of self-monitored decline in non-invasive estimated VO_2_ max (a feature in Apple Watch) that reflected progression of severity of aortic stenosis, which led to timely transcatheter valvular intervention.

## Case presentation

A 68-year-old male with hypertension and hyperlipidaemia was referred in 2022 for evaluation of a murmur. He denied exertional shortness of breath, chest pain, or syncope and reported running daily without limitations. Baseline transthoracic echocardiography (TTE) from 2019 revealed moderate AS with an AVA of 1.3 cm^2^, peak velocity 3.2 m/s, and mean transaortic pressure gradient of 23 mmHg.

**Figure 1:**
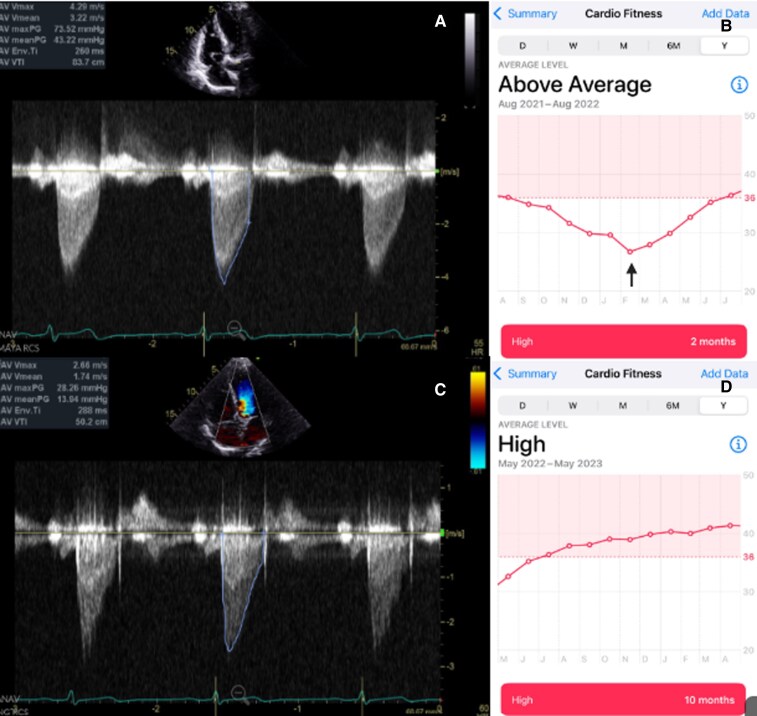
(*A*) Continuous-wave Doppler echocardiography shows high transvalvular velocity (4.29 m/s) and mean gradient of 43 mmHg, consistent with severe aortic stenosis. (*B*) Apple Watch cardiofitness application: estimated VO_2_ max trend showing progressive decline from approximately 36–26 mL/kg/min over months preceding TAVR. The nadir point (arrow) corresponds to the time of documented haemodynamic progression. X axis: time in months; Y axis: Apple Watch–estimated VO_2_ max in mL/kg/min. (*C*) Post-TAVR echocardiogram demonstrating markedly reduced transvalvular velocity (2.66 m/s) with a mean gradient of 13 mmHg, confirming relief of obstruction. (*D*) Subsequent Apple Watch–derived VO_2_ max trend demonstrating steady recovery and normalization of estimated VO_2_ max (∼41 mL/kg/min) during follow-up after valve replacement.

Over the following 3 years, he continued regular aerobic exercise and tracked this Apple Watch–derived VO_2_ max, which decreased from 36 to 26 mL/kg/min (*Figure 1B*). Concerned by the trend, he sought our evaluation. Transthoracic echocardiography in our office demonstrated a severely calcified aortic valve with AVA 0.6 cm^2^, mean gradient was 43 mm Hg, and peak velocity was 4.29 m/s with a normal LVEF 60%–65% (*Figure 1A*). The patient was asymptomatic and noted decrease in exercise capacity with his documented Apple Watch MVO_2_. Based on the multidisciplinary heart team evaluation and patient’s preference, transcatheter aortic valve replacement (TAVR) was performed using 26 mm Edwards Sapien 3 Ultra bioprosthesis without any complications.

At 1-month, 6-month, and 1-year follow-up status post-TAVR, the patient had resumed baseline physical activity, with VO_2_ max increasing progressively to 41 mL/kg/min (*Figure 1D*). Echocardiography at 3-year follow-up post-TAVR showed a well-seated bioprosthetic valve with the mean gradient of 9 mm Hg and no paravalvular leak (*Figure 1C*).

## Discussion

This case highlights the potential of wearable technology as an adjunctive tool for monitoring disease progression in valvular heart diseases. Estimated VO_2_ max, derived from heart rate and accelerometry data, provides a measure of cardiopulmonary fitness and correlates reasonably well with laboratory measured MVO_2_.^1,2^

In AS, objective assessment of exercise tolerance is essential to determining the timing of intervention, especially in apparently asymptomatic patients. Current guidelines recommend exercise testing to unmask symptoms or abnormal haemodynamic responses, yet such testing is infrequently performed.^3^ The observed decline in estimated VO_2_ max proceeded the onset of symptoms in this patient correlates with the echocardiographic progression of severe AS. Our patient fulfilled criteria per 2020 ACC/AHA guidelines for asymptomatic severe aortic stenosis with rapid disease progression based on significant decline in surrogate MVO_2_ serial measurements and severe aortic valve calcification on echocardiography.^3^ The patient declined further stress testing and preferred TAVR as therapeutic intervention.

Wearable technology, such as Apple Watch, offers novel, continuous, and patient-centred means of physiologic surveillance.^2^ Integrating these data into clinical follow-up may improve the detection of functional decline and enable timely intervention before decompensation occurs. The estimated VO_2_ max trended steadily downward over several months from 36 to 26 mL/kg/min despite unchanged exercise habits and absence of subjective limitation. This may be explained by increasing fixed obstruction due to progressive valvular calcification and a reduced capacity to augment cardiac output during exertion.

Despite its promising role, the use of wearable-derived VO_2_ max has several limitations. First, the Apple Watch algorithm for VO_2_ max estimation is proprietary and not specifically validated in patients with cardiovascular disease, arrhythmias, or valvular abnormalities. Motion artefacts, skin tone, and device positioning can all influence readings.^2^ Furthermore, VO_2_ max estimation assumes consistent effort and gait mechanics, which may not reflect actual physiologic capacity in individuals with changing haemodynamics or medication use (such as beta-blockers). Large perspective studies correlating wearable-derived data with echocardiographic parameters and exercise testing are needed to define clinical thresholds for intervention.

## Data Availability

Data supporting this case report are available from the corresponding author upon reasonable request.

## Lead author biography

The lead author is Dr Mukhtar Al-Saadi who is currently a chief cardiovascular diseases fellow at HCA Houston, TX, USA, affiliated with University of Houston. He did his internal medical residency training at Detroit Medical Medical, Detroit, MI, USA, then further training in critical care medicine at Baylor College of Medicine in Houston, TX, USA.

## Notes

**Consent:** The patient authorized the use and publication of their de-identified medical information (case details and images) for teaching or research, acknowledging efforts for anonymity, but the possibility of identification and understanding their participation is voluntary and does not affect care. The patient provided full consent in compliance with COPE guidelines.
